# Cadmium-inducible expression of the ABC-type transporter *AtABCC3* increases phytochelatin-mediated cadmium tolerance in *Arabidopsis*

**DOI:** 10.1093/jxb/erv185

**Published:** 2015-04-21

**Authors:** Patrizia Brunetti, Letizia Zanella, Angelo De Paolis, Davide Di Litta, Valentina Cecchetti, Giuseppina Falasca, Maurizio Barbieri, Maria Maddalena Altamura, Paolo Costantino, Maura Cardarelli

**Affiliations:** ^1^Istituto di Biologia e Patologia Molecolari, CNR, Sapienza Università di Roma, Rome, Italy; ^2^Dipartimento di Biologia e Biotecnologie, Sapienza Università di Roma, Rome, Italy; ^3^Dipartimento di Biologia Ambientale, Sapienza Università di Roma, Rome, Italy; ^4^Istituto di Scienze delle Produzioni Alimentari, CNR, Lecce, Italy; ^5^Dipartimento di Scienze della Terra, Sapienza Università di Roma, Rome, Italy

**Keywords:** ABC-type transporters, *Arabidopsis*, cadmium stress, cadmium tolerance, phytochelatins, vacuolar compartmentalization.

## Abstract

The heavy metal cadmium (Cd) is a widespread environmental contaminant with harmful effects on living cells. In plants, phytochelatin (PC)-dependent Cd detoxification requires that PC–Cd complexes are transported into vacuoles. Here, it is shown that *Arabidopsis thaliana* seedlings defective in the ABCC transporter AtABCC3 (*abcc3*) have an increased sensitivity to different Cd concentrations, and that seedlings overexpressing *AtABCC3* (AtABCC3ox) have an increased Cd tolerance. The cellular distribution of Cd was analysed in protoplasts from *abcc3* mutants and *AtABCC3* overexpressors grown in the presence of Cd, by means of the Cd-specific fluorochromes 5-nitrobenzothiazole coumarin (BTC-5N) and Leadmium™ Green AM dye. This analysis revealed that Cd is mostly localized in the cytosol of *abcc3* mutant protoplasts whereas there is an increase in vacuolar Cd in protoplasts from AtABCC3ox plants. Overexpression of *AtABCC3* in *cad1-3* mutant seedlings defective in PC production and in plants treated with l-buthionine sulphoximine (BSO), an inhibitor of PC biosynthesis, had no effect on Cd tolerance, suggesting that *AtABCC3* acts via PCs. In addition, overexpression of *AtABCC3* in *atabcc1 atabcc2* mutant seedlings defective in the Cd transporters AtABCC1 and AtABCC2 complements the Cd sensitivity of double mutants, but not in the presence of BSO. Accordingly, the level of *AtABCC3* transcript in wild type seedlings was lower than that of *AtABCC1* and *AtABCC2* in the absence of Cd but higher after Cd exposure, and even higher in *atabcc1 atabcc2* mutants. The results point to AtABCC3 as a transporter of PC–Cd complexes, and suggest that its activity is regulated by Cd and is co-ordinated with the activity of AtABCC1/AtABCC2.

## Highlight

AtABCC3 detoxifies cadmium by transporting phytochelatin–cadmium complexes into the vacuoles, and it can functionally complement *abcc1 abcc2* mutants.

## Introduction

Cadmium (Cd) is a heavy metal that exerts a detrimental effect on plants and on human health by interfering with biochemical functions of essential metals. Higher plants respond to Cd exposure by producing phytochelatins (PCs), cysteine-rich peptides with the general structure (Glu–Cys) *n*-Gly, where *n* is in the range of 2–11 (Grill *et al.*, 1985; Rauser, 1990). PCs also protect plants from the toxic effects of other heavy metals/metalloids such as lead (Pb), mercury (Hg), and arsenic (As), and have also been identified in the majority of algae, in fungi, including *Schizosaccharomyces pombe*, and in the worm *Caenorhabditis elegans* (Ha *et al.*, 1999). PCs are synthesized by phytochelatin synthase (PCS) from the substrate glutathione (GSH) (Grill *et al.*, 1989; Thangavel *et al.*, 2007), and *PCS* genes were first isolated from *Arabidopsis thaliana*, *S. pombe*, *Triticum aestivum*, and *C. elegans* (Ha *et al.*, 1999; Clemens *et al.*, 1999, 2001; Vatamiunik *et al.*, 1999; Cobbett, 2000a, *b*). Subsequently *PCS* genes have been isolated from different plants such as *Brassica juncea* (Heiss *et al.*, 2003) and invertebrate species such as the slime mould *Dictyostelium discoideum* (Cobbett, 2000a).

PCs are able to bind cytoplasmic Cd, forming stable PC–Cd complexes, playing a major role in Cd detoxification: PC-deficient mutants of *S. pombe* and *Arabidopsis*—*cad1*, mutated in *AtPCS1*—are hypersensitive to Cd (Ha *et al.*, 1999); accordingly, in most species, *PCS* overexpression leads to increased Cd tolerance (Vatamaniuk *et al.*, 1999; Gisbert *et al.*, 2003; Sauge-Merle *et al.*, 2003; Martinez *et al.*, 2006; Pomponi *et al.*, 2006; Gasic and Korban, 2007; Guo *et al.*, 2008; Wojas *et al.*, 2010; Brunetti *et al.*, 2011). The mechanism of detoxification mediated by PCs requires that PC–Cd complexes are transported by specific proteins into the vacuoles where they form more stable high molecular weight complexes by sulphide bonds. In addition, Cd can be transported directly into vacuoles by vacuolar Ca^2+^/H^+^ antiporters (Salt and Wagner, 1993; Clemens *et al.*, 2001). Early experiments on isolated vacuoles from *Avena sativa* roots suggested that transport of PC–Cd complexes is mediated by ATP-binding cassette (ABC)-type transporters (Salt and Rauser, 1995), ubiquitous transmembrane proteins that utilize ATP to translocate various substrates across membranes. ABC proteins have a characteristic modular structure consisting of a double set of two basic structural elements, a hydrophobic transmembrane domain (TMD) usually made up of six membrane-spanning α-helices, and a cytosolic domain containing a nucleotide-binding domain (NBD) involved in ATP binding (Wanke and Kolukisaoglu, 2010); the two TMDs dimerize to form the substrate-binding cavity (Procko *et al.*, 2009). The first protein that has been assigned a role as a PC–Cd vacuolar transporter is the half ABC transporter molecule HMT1 (HEAVY METAL TOLERANCE-FACTOR1) in *S. pombe*; this transporter, which has only one NBD and one TMD, needs to homo- or heterodimerize to become functional (Ortiz *et al.*, 1995). Subsequently, *HMT1* homologues have been identified in *C. elegans* (Vatamaniuk *et al.*, 2005) and in *Drosophila melanogaster* (Sooksa-Nguan *et al.*, 2009), but not in higher plants. More recently, an ABCC-type transporter Abc2 (belonging to the ABCC/MRP subfamily of ABC transporters) has been identified as the main PC–Cd transporter in *S. pombe* (Mendoza-Cózatl *et al.*, 2010). On the other hand, it has been shown that in *Saccharomyces cerevisiae*, which lacks PCS and does not produce PCs, the ABCC-type transporter YCF1 is able to transport GSH–Cd complexes into the vacuole (Li *et al.*, 1997), and overexpression of *ScYCF1* increases Cd tolerance in *Arabidopsis* seedlings (Song *et al.*, 2003).

In *Arabidopsis*, the ABCC family consists of 15 ABC proteins, characterized by the presence of an additional N-terminal TMD (TMD0) of unknown function (Klein *et al.*, 2006), although it has been shown that in some human and yeast ABCCs TMD0 is involved in protein targeting. Most ABCC proteins are localized in the vacuolar membrane and have been considered good candidates as transporters of PC–heavy metal complexes. In particular, AtABCC3, AtABCC4, and AtABCC7 when expressed individually in *S. cerevisiae* are able to complement the loss of YCF1, partially restoring Cd tolerance (Klein *et al.*, 2006). Very recently, it has been shown that AtABCC1 and AtABCC2—first identified as transporters of PC–As complexes—play a role in Cd (and Hg) tolerance (Park *et al.*, 2012). However, it has not yet been established whether AtABCC3, which is also up-regulated by Cd treatment together with AtABCC6 and AtABCC7 (Gaillard *et al.*, 2008), also plays a role in PC-mediated Cd detoxification. Here, by analysis of Cd tolerance of *abcc3* knockout mutants defective in *AtABCC3*, and by *AtABCC3* overexpression in wild type, PC-deficient lines, and *atabcc1 atabcc2* double mutants, combined with analysis of cellular Cd localization, and comparative analysis of Cd tolerance between *abcc3* and *atabcc1 atabcc2* double mutants, it is shown that AtABCC3 is involved in the vacuolar transport of PC–Cd complexes.

## Material and methods

### Plant growth conditions and metal treatments

Wild type, mutant lines *abcc3* (kindly provided by Markus Klein of Philip Morris International, Switzerland), *abcc1 abcc2* (Song *et al.*, 2010), kindly provided by Enrico Martinoia (University of Zurich, Switzerland), and *cad1-3* (Cobbett, 2000a; kindly provided by Chris Cobbett of University of Melbourne, Australia) AtPCSox-21, AtPCSox-20, AtPCSox-26, AtABCC3ox-*cad1*-53, AtABCC3ox-*cad1*-59, AtABCC3ox-*abcc1abcc2*-1, AtABCC3ox-*abcc1abcc2*-3, and AtABCC3ox-*abcc1abcc2*-5 seedlings were germinated on half-strength Murashige and Skoog (MS) basal agar medium (pH 5.8) (Murashige and Skoog, 1962) in a growth chamber in a 16/8h light/dark cycle at 22 °C. After 7 d, 10 seedlings were transferred to a half-strength MS basal medium with 0.5% sucrose, at different concentrations of CdSO_4_ (0, 15, 30, 60, and 90 μM) in the presence of 10 μM β-oestradiol when indicated. Seedling fresh weight and root length were measured after 5 d or 9 d of further growth.

To assess the effect of l-buthionine sulphoximine (BSO) on Cd sensitivity, 7-day-old seedlings were transferred to medium containing 60 μM CdSO_4_ with or without 0.5mM BSO. Seedling fresh weight and root length were measured after 9 d of further growth. The experiments were performed in triplicate.

To analyse Cd content, two experiments were performed as follows. (i) Seven days after germination, ~50 seedlings for each plant were placed into holes of a plastic septum in a phytatray (Sigma), so that only roots were immersed in liquid medium. A half-strength MS medium (0.5% sucrose) was supplemented with 10 μM β-oestradiol, and 60 μM CdSO_4_ was added. Seedlings, shaken occasionally, were harvested after 9 d. (ii) Seven days after germination, ~130 seedlings for each line were transferred to a half-strength MS basal medium with 0.5% sucrose, at 30 μM or 60 μM CdSO_4_ in the presence of 10 μM β-oestradiol. Seedlings were harvested after 2 weeks. The experiments were performed in triplicate.

### Plant expression construct, transformation, and selection

An XbaI–XbaI fragment harbouring the coding region of *AtABCC3* was cloned into the *Spe*I site of the binary plasmid pER8, under the control of an oestrogen-inducible promoter (Zuo *et al.*, 2000). *Agrobacterium tumefaciens* strain GV3101 carrying the construct *pER8::35S-ABCC3* was used to transform *A. thaliana* wild type plants (ecotype Columbia) by standard floral dip transformation (Clough and Bent, 1998). Transformed plants were analysed by PCR with the following primers: LexA 4096 For 5′-GCCATGTAATATGCTCGACT-3′, MRP3 Rev 4467 5′-GAGCTGACTTAAACCCAAAAT-3′; and by real-time reverse tanscription–PCR (RT–PCR; see below). Homozygous T_2_ generations were obtained by self-fertilization of primary transformants and the seeds were grown as described below (Cecchetti *et al.*, 2008).

### Quantitative RT–PCR analysis

RNA was extracted from 50mg of seedlings grown at the indicated CdSO_4_ concentration in the presence or absence of the inducer β-oestradiol and reverse-transcribed as previously described. SYBR Green-based quantitative assays were performed using a Bio-Rad iCicler iQ as described in Cecchetti *et al.* (2013). The primers used to analyse *AtABCC3* transcript levels were: RTmrp3 For 3835 5′-CTTCAGGTCCGATATGCTCCA-3′, RTmrp3 Rev 3885 5′-TGTTATTCCTCGCAACACAAGAG-3′; ACTIN2 For 5′-CCGATCCAGACACTGTACTTCCTT-3′, ACTIN2 Rev 5′-CTTGCACCAAGCAGCATGAA-3′, and were designed as previously described (Cecchetti *et al.*, 2004). The experiments were performed in triplicate.

### Cross-pollination

Homozygous *cad1-3* lines were used for crosses with homozygous AtABCC3ox-21 lines. F_2_ lines, homozygous for the AtABCC3ox construct and for the *cad1-3* mutation, were selected on hygromycin, and the *cad1-3* mutation was verified by PCR with the following primers: *cad1-3* For 5′-TCAAGTATCCCCCTCACTGC-3′; *PCS1* For 5′-TCAAGTATCCCCCTCACTGG-3′; and *PCS1* Rev 5′-CGGGTTCTCTGTGTGGTCTA-3′. Three independent homozygous lines named AtABCC3ox-20, AtABCC3ox-21, and AtABCC3ox-26 were used for subsequent Cd tolerance analysis.

### Statistical analysis

Two-tailed and one-tailed Student’s *t*-tests were used to evaluate statistical significance. All the statistical analyses were performed using Graph Pad Prism 5 (Graph Pad Software Inc.).

### Intracellular Cd localization through Cd-sensing fluorescent dyes

Wild type, *abcc3*, and AtABCC3ox seedlings were grown on half-strength MS agarized medium in the absence or presence of 60 μM CdSO_4_. β-Oestradiol (10 μM) was added in experiments conducted with AtABCC3ox lines when indicated. Leaf protoplasts were prepared from wild type and *abcc3* plants after 9 d or 22 d of treatment, whereas they were prepared from AtABCC3ox lines after 5 d and 9 d of treatment. The enzymatic digestion was carried according to Lindberg *et al.* (2004). The same number of isolated protoplasts from wild type, *abcc3*, and AtABCC3ox were loaded either with 0.5% 5-nitrobenzothiazole coumarin (BTC-5N) (Lindberg *et al.*, 2004) in dimethylsulphoxide (DMSO)/pluronic aqueous solution (Molecular Probes, Leiden, The Netherlands) or with 0.5% Leadmium™ Green AM dye (Molecular Probes, Invitrogen, Carlsbad, CA, USA) in DMSO, and treated as described. The fluorescence signal was observed using a DMRB microscope equipped with a specific filter sets (excitation at 415nm and emission at 500–530nm for BTC-5N, and excitation at 484/15nm and emission at 517/30nm for Leadmium™ Green AM dye). Images were acquired with a LEICA DC500 digital camera and analysed with the IM1000 image-analysis software (Leica). Regions inside the vacuole and within the cytosol were selected from 30 single protoplast images per genotype and the mean intensity value of the epifluorescence was quantified using the ImageJ 1.36 b analysis software (National Institute of Health, Bellevue, WA, USA) and expressed in arbitrary units (AUs; from 0 to 255). The experiment was repeated three times; data from one experiment were reported.

### Cadmium accumulation through ICP-MS analysis

Wild type, AtABCC3ox-26, and AtABCC3ox-21 seedlings, cultured as described above, were washed with distilled water—with shoots and roots separated when necessary—and dried at 80 °C overnight. Dried tissues were weighed and then ground in a mortar. Homogenized material was mineralized in a microwave oven (Milestone Ethos 1600) with HNO_3_ and H_2_O_2_ (3:1) under high temperature and pressure. Mineralized samples were analysed for total Cd detection, using inductively coupled plasma-mass spectrometry (ICP-MS; ThermoFisher Serie II). All analyses were performed in three replicates. The amounts of acids used were the same as the amounts of additives in the digested samples in the digestion batch. Analytical accuracy was determined using certiﬁed reference material of the Community Bureau of Reference.

## Results

### Cd tolerance is decreased in *abcc3* mutants and enhanced in *AtABCC3* overexpressors

**Fig. 1.**
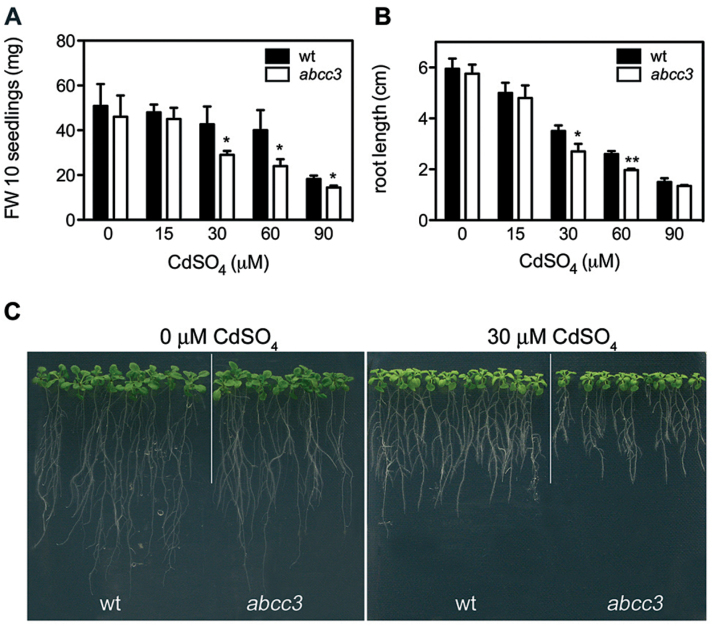
Cd tolerance of wild type and *abcc3* seedlings. (A, B) Wild type and *abcc3* seedlings were incubated on medium containing 0, 15, 30, 60, or 90 μM CdSO_4_. (A) Fresh weight and root length (B) were measured after 9 d. (C) Wild type and *abcc3* seedlings at 0 and 30 μM CdSO_4_. Values correspond to means (*n*=3). Error bars indicate the SE. Asterisks indicate a significant difference from the wild type (*P<0.05, **P<0.01). wt, wild type.

To assess whether *AtABCC3* contributes to Cd tolerance, the growth of wild type and *abcc3* seedlings was analysed at different Cd concentrations. In a previous study, it was shown that growth of *Arabidopsis* seedlings is not affected at Cd concentrations up to 15 μM, while it is slightly reduced at 30 μM and 60 μM CdSO_4_, and severely inhibited at 90 μM (Brunetti *et al.*, 2011). Here, 7 d after germination, wild type and *abcc3* seedlings were grown in the presence of 0, 15, 30, 60, and 90 μM CdSO_4_, and the fresh weight and root length were analysed after 9 d. As shown in Fig. 1, in the absence of Cd and at 15 μM CdSO_4_, the growth of *abcc3* and wild type seedlings was comparable, whereas in the presence of all Cd concentrations from 30 μM onwards the former was slightly but significantly more inhibited than the latter (Fig. 1A–C). In terms of fresh weight, the growth of *abcc3* seedlings was inhibited from a concentration of 30 μM CdSO_4_ onwards (Fig. 1A–C), whereas roots were significantly shorter only at 30 μM and 60 μM CdSO_4_ (Fig. 1B, C).

**Fig. 2.**
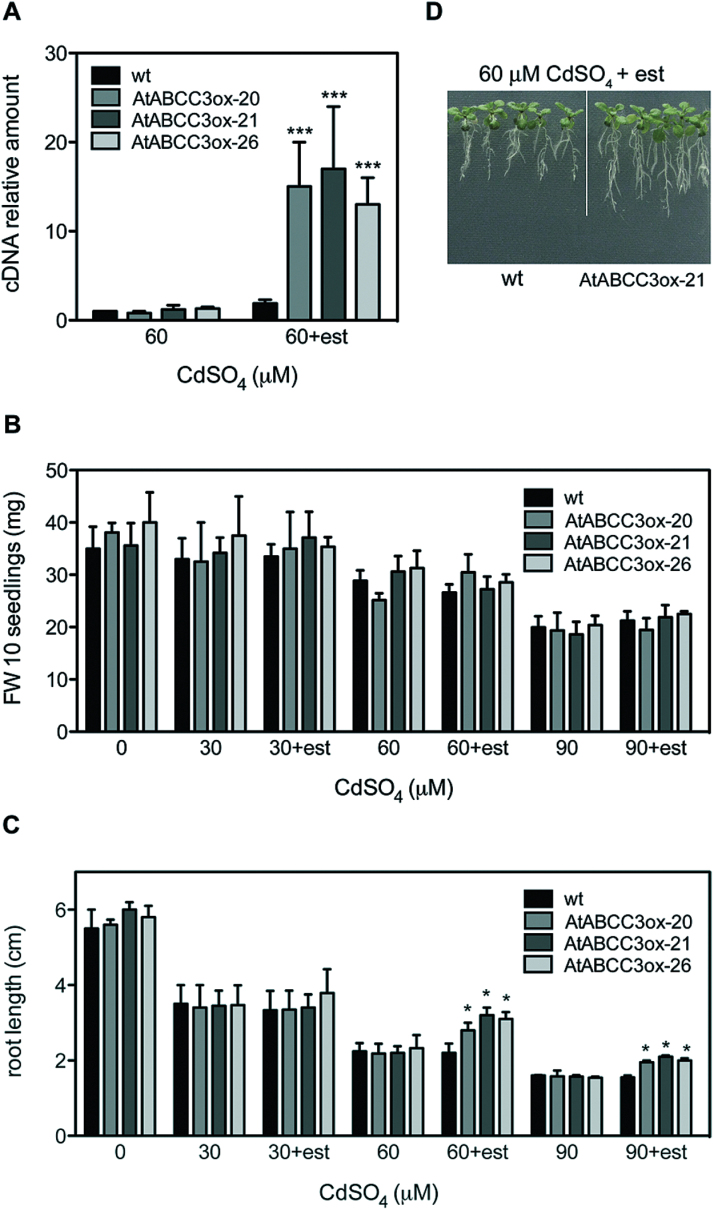
Quantitative analysis of *AtABCC3* transcript and Cd tolerance of wild type and AtABCC3ox seedlings. (A) Real-time RT–PCR of mRNA extracted from wild type, AtABCC3ox-20, AtABCC3ox-21, and AtABCC3ox-26 seedlings grown for 9 d at 60 μM CdSO_4_, in the absence or presence of β-oestradiol. Data are expressed as a mean value (*n*=3) of *AtABCC3* cDNA levels relative to actin cDNA. Error bars indicate the SE. (B, C) Wild type, AtABCC3ox-20, AtABCC3ox-21, and AtABCC3ox-26 seedlings were incubated on medium containing 0, 30, 60, or 90 μM CdSO_4_ in the absence or presence of β-oestradiol. (B) Fresh weight and root length (C) were measured after 9 d. (D) AtABCC3ox-21 seedlings compared with wild type seedlings after 9 d at 60 μM CdSO_4_, in the presence of β-oestradiol. Values correspond to means (*n*=3). Error bars indicate the SE. Asterisks indicate a significant difference from seedlings grown in the absence of β-oestradiol (A) or a significant difference from wild type roots (C) (*P<0.05, ***P<0.001). est, β-oestradiol; wt, wild type.

These results suggest an involvement of *AtABCC3* in Cd tolerance, and, to confirm this notion, *Arabidopsis* lines overexpressing *AtABCC3* (AtABCC3ox) under the control of a β-oestradiol-inducible promoter were produced (Zuo *et al.*, 2000). Overexpression of *AtABCC3* was analysed by means of real-time RT–PCR (qRT-PCR) in three independent homozygous lines named AtABCC3ox-20, AtABCC3ox-21, and AtABCC3ox-26. Seedlings from the wild type and these AtABCC3ox lines were grown in the presence of 60 μM CdSO_4_ with or without the inducer β-oestradiol, and *AtABCC3* transcript levels were analysed after 9 d of growth. As shown in Fig. 2A, the *AtABCC3* mRNA level increased ~15-, 17-, and 13-fold compared with the wild type in AtABCC3ox-20, AtABCC3ox-21, and AtABCC3ox-26 seedlings, respectively.

An effect of β-oestradiol on seedling growth was ruled out, as no significant differences in fresh weight and root length were observed between wild type and AtABCC3ox seedlings after 9 d of growth in the presence or absence of β-oestradiol, without Cd (Supplementary Fig. S1 available at *JXB* online).

To assess Cd tolerance, AtABCC3ox-20, AtABCC3ox-21, and AtABCC3ox-26 seedlings were grown in the presence of 0, 30, 60, and 90 μM CdSO_4_ with or without β-oestradiol, and the fresh weight and root length were analysed after 9 d. No significant differences in either growth indicator were observed at 30 μM CdSO_4_ (Fig. 2B, C) in any of the AtABCC3ox seedlings grown in the presence or absence of the inducer. At 60 μM CdSO_4_, all three AtABCC3ox lines showed a significant increase in root length when grown in the presence of the inducer (Fig. 2C, D), whereas the fresh weight was comparable in seedlings grown in the presence or absence of β-oestradiol (Fig. 2B).

At 90 μM CdSO_4_, all three AtABCC3ox lines showed a significant increase in root length (Fig. 2C), but not in fresh weight when grown in the presence of the inducer (Fig. 2B).

These results confirm an involvement of *AtABCC3* in Cd tolerance.

### *AtABCC3* is involved in vacuolar Cd^2+^ sequestration

To determine whether AtABCC3 plays a role in Cd transport into the vacuole, the cellular distribution of Cd was compared in the wild type and *abcc3* mutants by means of selective Cd-sensing fluorochromes: BTC-5N (Lindberg *et al.*, 2004, 2007) and Leadmium™ Green AM dye (Lu *et al.*, 2008), specific for cytosolic and vacuolar Cd accumulation, respectively. Wild type protoplasts have been preliminarily used to define the cytosolic and vacuolar regions independently of the fluorescence, as shown in Supplementary Fig. S2 at *JXB* online. Leaf protoplasts were isolated from wild type and *abcc3* plants grown in the absence or presence of 60 μM CdSO_4_ for 9 d and 22 d, and loaded with either one of the two fluorochromes.

**Fig. 3.**
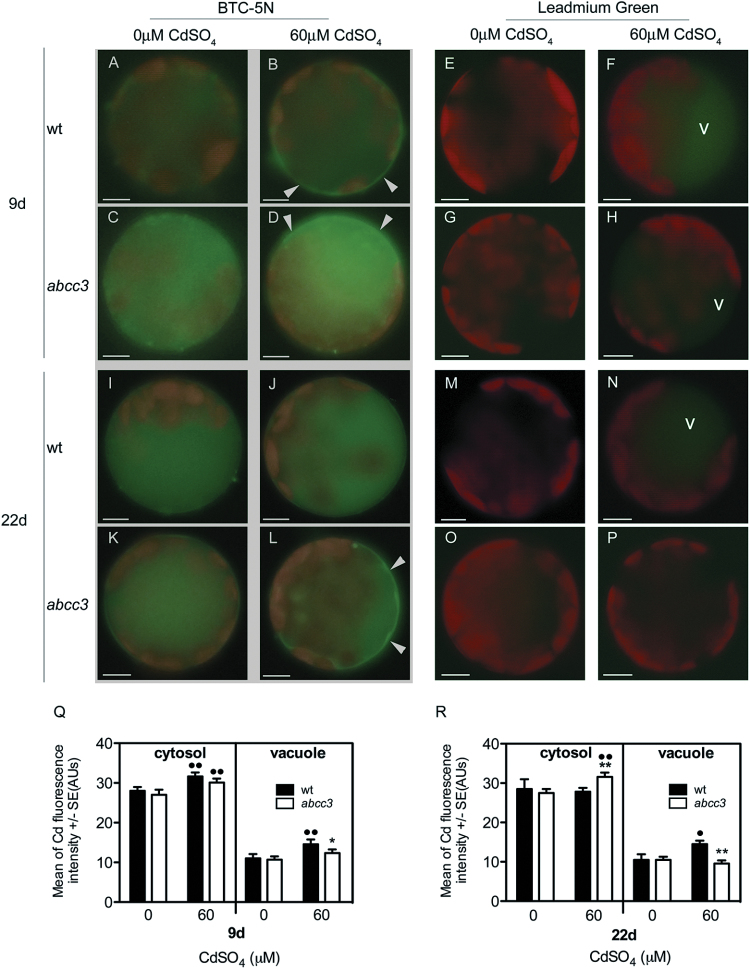
Analyses of cytosolic and vacuolar Cd in wild type and *abcc3* leaf protoplasts. Protoplasts from wild type and *abcc3* mutant plants grown in the absence or presence of 60 μM CdSO_4_ were loaded with the cytosolic Cd-sensing fluorochrome BTC-5N and the vacuolar Cd-sensitive probe Leadmium™ Green AM dye. (A–D, I–L) Fluorescent images of protoplasts loaded with BTC-5N at 9 d and 22 d, respectively. Wild type (A, I) and *abcc3* (C, K) protoplasts from plants grown in the absence of Cd for 9 d or 22 d. Wild type (B, J) and *abcc3* (D, L) protoplasts from plants grown in the presence of 60 μM CdSO_4_ for 9 d or 22 d. The Cd-specific cytosolic fluorescence signal is indicated by arrows (B, D, L). (E–H, M–P) Fluorescent images of protoplasts loaded with Leadmium™ Green AM dye at 9 d and 22 d, respectively. Wild type (E, M) and *abcc3* (G, O) protoplasts from plants grown in the absence of Cd for 9 d or 22 d. Wild type (F, N) and *abcc3* (H, P) protoplasts from plants grown in the presence of 60 μM CdSO_4_ for 9 d and 22 d. (Q, R) Fluorescence signal intensity in the cytosol and in the vacuole of wild type and *abcc3* protoplasts from plants grown in the absence or presence of 60 μM CdSO_4_ for 9 d and 22 d. Values are means (*n*=30). Error bars indicate the SE. Asterisks indicate a significant difference from wild type protoplasts (*P <0.05, **P<0.01). Dots indicate a significant difference between Cd treatment and control within the same genotype (•*P*<0.05, ••*P*<0.01). V, vacuole; wt, wild type. Scale bars=10 μm.

As shown in Fig. 3, BTC-5N-loaded protoplasts isolated from wild type and *abcc3* plants grown in the absence of CdSO_4_ exhibited an orange-green signal due to red chlorophyll autofluorescence, and a green signal due to complexes between the fluorochrome and cytosolic divalent ions other than Cd (Fig. 3A, C, I, K, Q, left panel). When wild type and *abcc3* plants were cultured in the presence of Cd, after 9 d BTC-5N-loaded protoplasts showed a comparable Cd-specific cytosolic fluorescence signal (Fig. 3B, D, Q, left panel), whereas after 22 d the Cd-specific cytosolic signal decreased in wild type protoplasts but significantly increased in *abcc3* protoplasts (*P*<0.01) (Fig. 3J, L, Q, left panel).

**Fig. 4.**
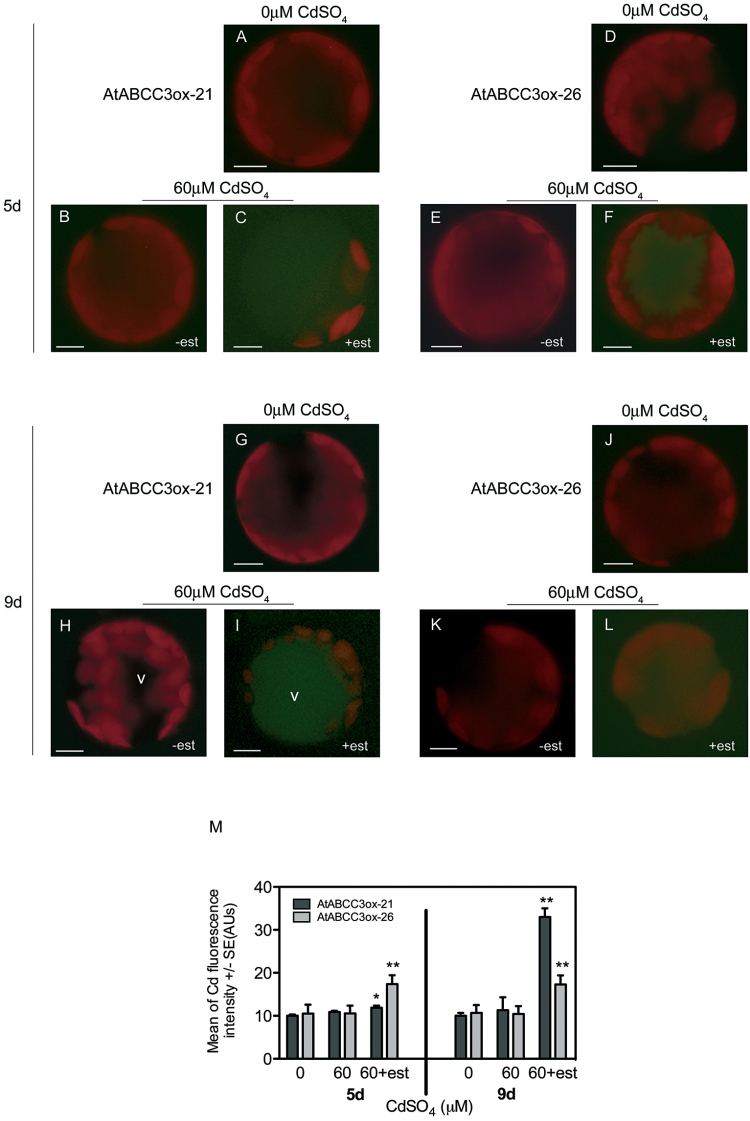
Analyses of vacuolar Cd in AtABCC3ox leaf protoplasts. Protoplasts from AtABCC3ox-21 and AtABCC3ox-26 overexpressing plants grown in the absence or presence of 60 μM CdSO_4_ with or without the inducer β-oestradiol were loaded with the vacuolar Cd-sensitive probe, Leadmium™ Green AM dye. (A–L) Fluorescent images of protoplasts loaded with Leadmium™ Green AM dye. AtABCC3ox-21 (A, G) and AtABCC3ox-26 (D, J) protoplasts from plants grown in the absence of Cd for 5 d or 9 d. AtABCC3ox-21 (B, C, H, I) and AtABCC3ox-26 (E, F, K, L) protoplasts from plants grown in the presence of 60 μM CdSO_4_ and β-oestradiol for 5 d and 9 d. (M) Fluorescence signal intensity in the vacuole of AtABCC3ox-21 and AtABCC3ox-26 protoplasts from plants grown in the absence or presence of 60 μM CdSO_4_ with or without β-oestradiol for 5 d and 9 d. Values are means (*n*=30). Error bars indicate the SE. Asterisks indicate a significant difference from the absence of β-oestradiol, within the same genotype (*P <0.05, **P<0.01). est, β-oestradiol; V, vacuole. Scale bars=10 μm.

Leadmium green-loaded protoplasts isolated from wild type and *abcc3* plants grown in the absence of Cd had a very low fluorescence signal that could be detected in the vacuole by quantitative analysis (see the Materials and methods) (Fig. 3Q, right panel, and Fig. 4M) but was not detectable in fluorescence images (Fig. 3E, G, M, O). This is possibly due to interactions between the fluorochrome and Ca^2+^ that occur in the absence of Cd. When wild type and *abcc3* protoplasts from plants cultured for 9 d in the presence of Cd were analysed, a slightly but significantly higher (*P*<0.05) fluorescence signal was detectable in the vacuoles of the former (Fig. 3F) than in those of the latter (Fig. 3H, Q, right panel). After 22 d in the presence of Cd, the vacuolar signal was almost unchanged in wild type vacuoles (Fig. 3N), whereas in vacuoles of *abcc3* protoplasts it became significantly lower (*P*<0.01) than in the wild type (Fig. 3P, Q, right panel).

These results indicate a decrease in vacuolar Cd and a concomitant increase in cytosolic Cd in *abcc3* mutant protoplasts compared with those of the wild type, suggesting a role for ABCC3 in Cd transport into the vacuole.

To confirm the involvement of ABCC3 in Cd compartmentalization, the vacuolar Cd signal was analysed in two different AtABCC3ox lines. To detect a possible increase in vacuolar Cd, AtABCC3ox-21 and AtABCC3ox-26 plants were grown in the presence of 0 and 60 μM CdSO_4,_ with or without β-oestradiol; leaf protoplasts isolated after 5 d or 9 d were loaded with Leadmium™ Green AM dye. After 5 d of treatment with Cd, protoplasts from AtABCC3ox-21 and AtABCC3ox-26 plants grown in the presence of β-oestradiol showed a significant increase (*P*<0.05 and *P*<0.01, respectively) in the vacuolar signal (Fig. 4C, F, M, left panel) compared with protoplasts grown without β-oestradiol (Fig. 4B, E). Analogously, after 9 d of treatment with CdSO_4_ in the presence of β-oestradiol, both AtABCC3ox-21 and AtABCC3ox-26 protoplasts exhibited a vacuolar signal (Fig. 4I, L, M, right panel) significantly higher (*P*<0.01) than that of protoplasts from plants grown without β-oestradiol (Fig. 4H, K).

**Fig. 5.**
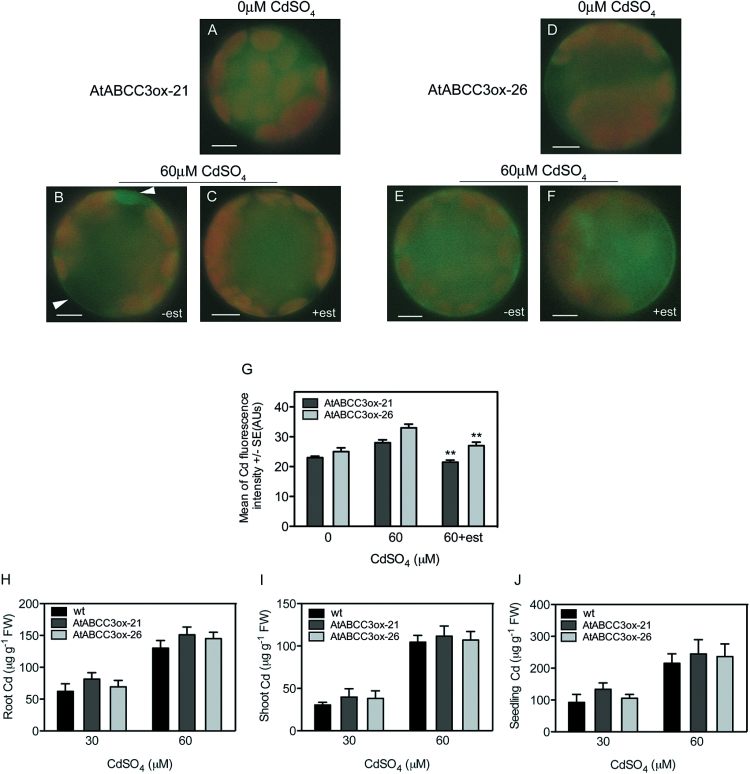
Analyses of cytosolic Cd in AtABCC3ox leaf protoplasts and of Cd content in AtABCC3ox shoots, roots, and seedlings. Protoplasts from AtABCC3ox-21 and AtABCC3ox-26 overexpressing plants grown in the absence or presence of 60 μM CdSO_4_, with or without the inducer β-oestradiol, were loaded with the cytosolic Cd-sensing fluorochrome BTC-5N. (A–F) Fluorescent images of protoplasts loaded with BTC-5N. AtABCC3ox-21 (A) and AtABCC3ox-26 (D) protoplasts from plants grown in the absence of Cd for 9 d. AtABCC3ox-21 (B, C) and AtABCC3ox-26 (E, F) protoplasts from plants grown in the presence of 60 μM CdSO_4_ with (C, F) or without (B, E) β-oestradiol for 9 d compared with those from plants grown without β-oestradiol. Cytosolic signal is indicated by arrows. (G) Fluorescence signal intensity in the cytosol of AtABCC3ox-21 and AtABCC3ox-26 protoplasts from plants grown in the absence or presence of Cd with or without β-oestradiol. Values are means (*n*=30). Error bars indicate the SE. Asterisks indicate a significant difference from the absence of β-oestradiol within the same genotype (**P<0.01). (H–J) Cd content in roots (H), shoots (I), and seedlings (J) of AtABCC3ox-21 and AtABCC3ox-26 seedlings overexpressing *AtABCC3* compared with wild type seedlings. Error bars indicate the SE (*n*=3). est, β-oestradiol. Scale bars=10 μm.

The Cd cytosolic signal was also analysed in protoplasts from AtABCC3ox-21 and AtABCC3ox-26 plants. After 9 d of treatment with Cd in the presence of β-oestradiol, AtABCC3ox-21 and AtABCC3ox-26 protoplasts exhibited a cytosolic signal (Fig. 5C, F, G) significantly lower (*P*<0.01) than that of protoplasts grown in the absence of β-oestradiol (Fig. 5B, E). These results indicate a lower cytosolic Cd accumulation and a corresponding increase in vacuolar Cd in AtABCC3ox protoplasts.

Taken together, these data on the cellular distribution of Cd in *abcc3* and in AtABCC3ox leaf protoplasts indicate that AtABCC3 plays an essential role in vacuolar cadmium sequestration.

To determine whether in ABCC3ox lines the increase in vacuolar Cd corresponds to an increase in total Cd accumulation, Cd content was analysed in wild type, AtABCC3ox-21, and AtABCC3ox-26 seedlings by means of ICP-MS. After 9 d of treatment with 60 μM CdSO_4_ in the presence of β-oestradiol, wild type, AtABCC3ox-21, and AtABCC3ox-26 seedlings showed a comparable content of total Cd (624±51.6, 696±12.22, and 698±40.01 μg g^–1^ FW, respectively).

To confirm these data, Cd content was analysed separately in shoots and roots from wild type, AtABCC3ox-21 and AtABCC3ox-26 seedlings exposed for 2 weeks at 30 μM or 60 μM CdSO_4._ As shown in Fig. 5, no significant difference in Cd content was observed at these Cd concentrations, in roots (Fig. 5H) or shoots (Fig. 5I), as well as in seedlings (Fig. 5J), of the overexpressing lines compared with the wild type. Together these data rule out an effect of *AtABCC3* overexpression on Cd accumulation.

### Overexpression of *AtABCC3* has no effect on Cd tolerance of seedlings lacking or with reduced PC synthesis

**Fig. 6.**
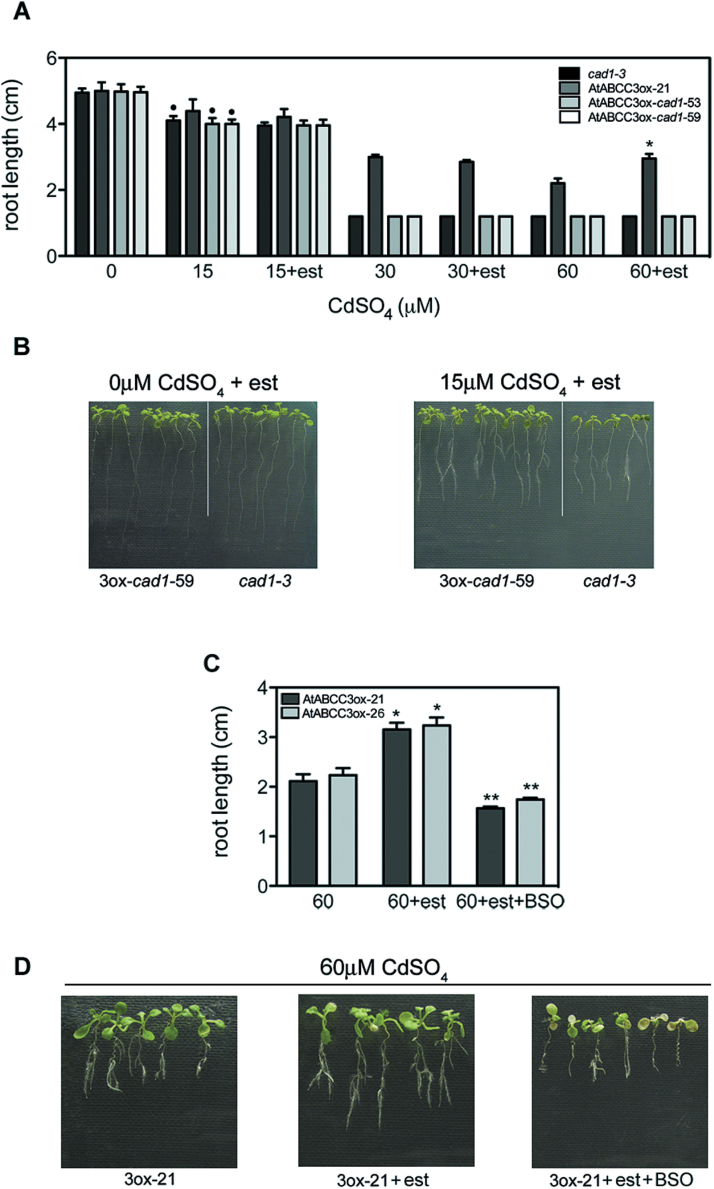
Cd tolerance of *cad1-3* seedlings overexpressing AtABCC3 and of AtABCC3ox seedlings in the presence of BSO. (A, B) *cad1-3*, AtABCC3ox-21, AtABCC3ox-*cad1*-53, and AtABCC3ox-*cad1*-59 were incubated on medium containing 0, 15, 30, or 60 μM CdSO_4_ in the absence or presence of β-oestradiol. (A) Root length was measured after 9 d. (B) AtABCC3ox-*cad1*-59 and *cad1-3* seedlings after 9 d at 0 and 15 μM CdSO_4_, in the presence of β-oestradiol. (C, D) AtABCC3ox-21 and AtABCC3ox-26 seedlings after 9 d at 60 μM CdSO_4_ with β-oestradiol in the presence and absence of BSO. (C) Root length was measured after 9 d. (D) AtABCC3ox-21 seedlings were incubated on medium containing 60 μM CdSO_4_ with or without β-oestradiol in the absence or presence of 0.5mM BSO. Values correspond to means (*n*=3). Error bars indicate the SE. est, β-oestradiol. Asterisks indicate a significant difference from roots grown in the absence of β-oestradiol (*P<0.05, **P<0.01). A single dot indicates a significant difference from roots grown in the absence of Cd within genotypes (•*P*<0.05). 3ox-21, AtABCC3ox-21; 3ox-*cad1*-59, AtABCC3ox-*cad1*-59.

To assess whether vacuolar sequestration of Cd by AtABCC3 is mediated by PCs, *AtABCC3* was overexpressed in a *cad1-3* mutant line defective in PCS and, consequently, in PC production (Howden *et al.*, 1995). AtABCC3ox-*cad1* plants were generated by crossing AtABCCox-21 with *cad1-3* lines, and *AtABCC3* overexpression was analysed in different lines homozygous for the *cad1* mutation and the AtABCC3ox construct. Two lines, AtABCC3ox-*cad1*-53 and AtABCC3ox-*cad1*-59, overexpressing *AtABCC3* in the presence of β-oestradiol (Supplementary Fig. S3A at *JXB* online) were used for subsequent analysis. To assess Cd tolerance, AtABCC3ox-*cad1*-53 and AtABCC3ox-*cad1*-59 seedlings together with seedlings of the two parental lines, *cad1-3* and AtABCC3ox-21, were grown in the presence of 0, 30, and 60 μM CdSO_4_ with or without β-oestradiol. After 9 d, the root length and fresh weight were analysed. As shown in Fig. 6A and Supplementary Fig. S3B at *JXB* online, in the absence of β-oestradiol at 30 μM and 60 μM CdSO_4_, *cad1-3*, AtABCC3ox-*cad1*-53, and AtABCC3ox-*cad1*-59 seedling growth was completely inhibited, whereas root length and fresh weight of AtABCC3ox-21 seedlings were comparable with those of wild type seedlings (Fig. 2B, C). In the presence of β-oestradiol (Fig. 6A; Supplementary Fig. S3B at *JXB* online) both concentrations of Cd were toxic to AtABCC3ox-*cad1*-53 and AtABCC3ox-*cad1*-59, pointing to a lack of effect of *AtABCC3* overexpression in growth rescue in the absence of PCs, and suggesting that AtABCC3 acts in concert with PCs to control Cd tolerance.

Interestingly, at 60 μM CdSO_4_, while AtABCC3ox-21 seedlings showed, as described above, a significant increase in root length upon addition of β-oestradiol (Figs 2C, 6A), the growth of *cad1-3*, AtABCC3ox-*cad1*-53, and AtABCC3ox-*cad1*-59 seedlings was unaffected by addition of the inducer (Fig. 6A; Supplementary Fig. S3B at *JXB* online). To determine whether *AtABCC3* overexpression enhances Cd tolerance at lower Cd concentrations that only slightly affect *cad1-3* seedling growth, AtABCC3ox-*cad1*-53, AtABCC3ox-*cad1*-59, *cad1-3*, and AtABCC3ox-21 seedlings were grown in the presence of 15 μM CdSO_4_ with or without the inducer β-oestradiol. As shown in Fig. 6A and B, after 9 d in the absence of β-oestradiol, AtABCC3ox-21 seedlings show a growth comparable with that without Cd, whereas *cad1-3*, AtABCC3ox-*cad1*-53, and AtABCC3ox-*cad1*-59 seedling growth was slightly but significantly inhibited in terms of root length (Fig. 6A). In the presence of the inducer, the growth of AtBCC3ox-21 seedlings is comparable with that of seedlings grown without the inducer or without Cd (Fig. 6A, B). More interestingly, when grown in the presence of β-oestradiol, AtABCC3ox-*cad1*-53 and AtABCC3ox-*cad1*-59 are as much inhibited in growth as the parent line *cad1-3* in terms of root length (Fig. 6A, B) but not of fresh weight (Supplementary Fig. S3B at *JXB* online).

To provide further evidence that the effect of AtABCC3 is mediated by PCs, Cd tolerance of AtABCC3ox-21 and AtABCC3ox-26 seedlings was assessed in the presence of BSO, an inhibitor of γ-glutamylcysteine synthetase (γ-GCS), an enzyme that modulates GSH and PC synthesis (Howden and Cobbett, 1992). AtABCC3ox-21 and AtABCC3ox-26 seedlings were grown at 60 μM CdSO_4_ with or without β-oestradiol, in the presence or absence of 0.5mM BSO, and root length was measured after 9 d. As shown in Fig. 6C and D, the increase in Cd tolerance observed in AtABCC3ox-21 and AtABCC3ox-26 seedlings when exposed to Cd in the presence of β-oestradiol was not observed when BSO was added to the medium.

These results indicate that when PC biosynthesis is abated or reduced, Cd severely affects *Arabidopsis* growth even when *AtABCC3* is overexpressed.

### *AtABCC3* contributes to Cd tolerance and its expression is regulated by Cd

**Fig. 7.**
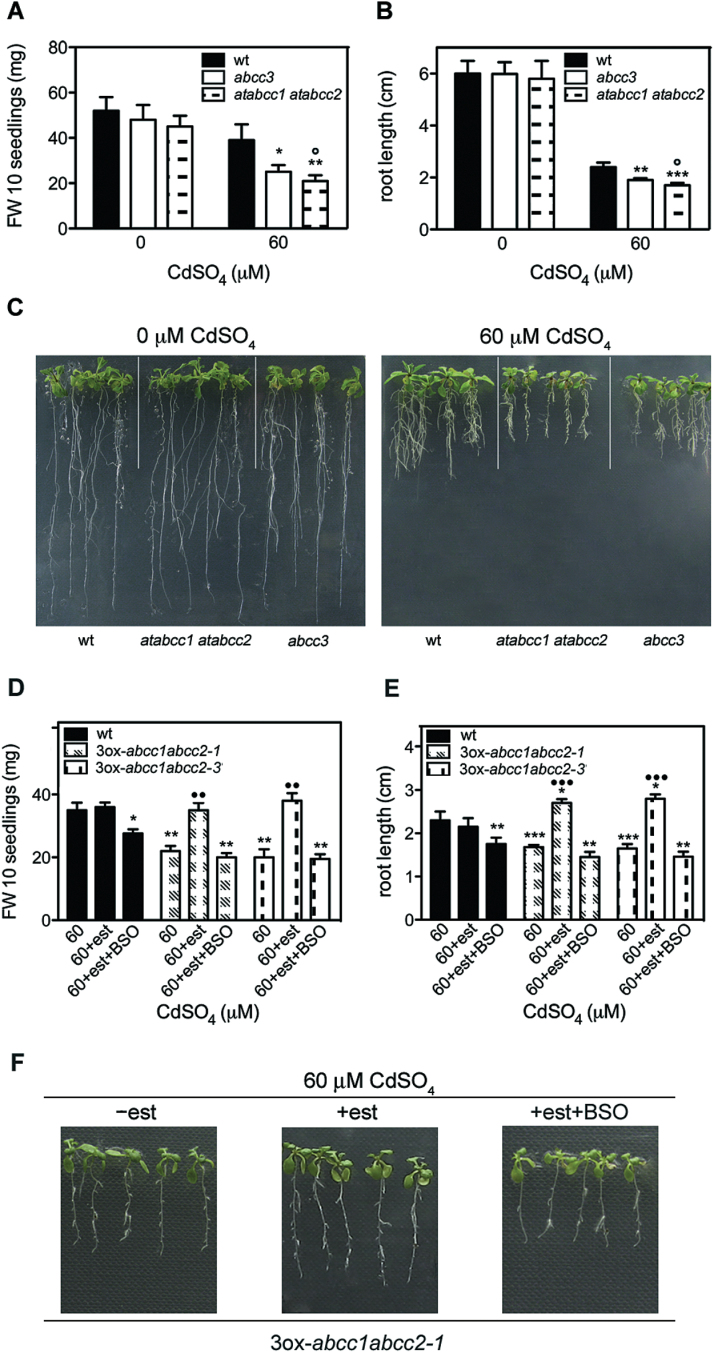
Comparative analysis of Cd tolerance of *abcc3* and *atabcc1 atabcc2* mutant seedlings and of *atabcc1 atabcc2* seedlings overexpressing AtABCC3 in the presence or absence of BSO. (A, B) Wild type, *abcc3*, and *atabcc1 atabcc2* seedlings were incubated on medium containing 0 and 60 μM CdSO_4_. (A) Fresh weight and root length (B) were measured after 9 d. (C) Wild type, *atabcc1 atabcc2*, and *abcc3* seedlings at 0 and 60 μM CdSO_4_. (D, E) Wild type, ABCC3ox-*atabcc1atabcc2*-1, and ABCC3ox-*atabcc1atabcc2*-3 seedlings were incubated on medium containing 60 μM CdSO_4_ with and without β-oestradiol, or with β-oestradiol in the presence of 0.5mM BSO. (D) Fresh weight and root length (E) were measured after 9 d. (F) ABCC3ox-*atabcc1atabcc2*-1 seedlings at 60 μM CdSO_4_ with (middle) and without β-oestradiol (left), or with β-oestradiol in the presence of 0.5mM BSO (right). Values correspond to means (*n*=3). Error bars indicate the SE. est, β-oestradiol. Asterisks indicate a significant difference from the wild type grown in the presence of 60 μM CdSO_4_ (*P<0.05, **P<0.01, ***P<0.001). A single circle indicates a significant difference from *abcc3* seedlings grown in the presence of 60 μM CdSO_4_ (°*P*<0.05). Dots indicate a significant difference from seedlings grown in the presence of 60 μM CdSO_4_ without β-oestradiol and BSO within the same genotype (••*P*<0.01, •••*P*<0.001). wt, wild type; 3ox-*abcc1abcc2*-1, AtABCC3ox-*abcc1abcc2*-1; 3ox-*abcc1abcc2*-3, AtABCC3ox-*abcc1abcc2*-3.

It has been reported that *AtABCC1* and, to a lesser extent, *AtABCC2* have a key role in Cd tolerance (Park *et al.*, 2012). To determine the contribution of *AtABCC3* to Cd tolerance relative to *AtABCC1* and *AtABCC2*, the growth of wild type, *abcc3*, and *atabcc1 atabcc2* double mutant seedlings was comparatively analysed at high Cd concentration (60 μM) where *AtABCC3* was shown to have an effect (see above). After 9 d in the absence of Cd the growth of wild type, *abcc3*, and *atabcc1 atabcc2* seedlings was comparable, whereas in the presence of 60 μM CdSO_4_ the growth of all seedlings was inhibited and, interestingly, *atabcc1 atabcc2* seedling growth was only slightly more inhibited than that of *abcc3* in terms of root length and fresh weight. This suggests a substantial contribution of AtABCC3 to Cd tolerance (Fig. 7A–C).

It was shown above that ABCC3 acts in the transport of PC–Cd complexes as do ABCC1 and ABCC2: it was thus asked whether ABCC3 could complement the *abcc1 abcc2* double mutation. To perform a complementation assay, *Arabidopsis abcc1 abcc2* lines overexpressing *AtABCC3* were produced by transforming *abcc1 abcc2* double mutant plants with the construct *pER8::35S-ABCC3* (see the Materials and methods). Overexpression of *AtABCC3* was measured by means of qRT-PCR in three independent homozygous lines denoted AtABCC3ox-*abcc1abcc2*-1, AtABCC3ox-*abcc1abcc2*-3, and AtABCC3ox-*abcc1abcc2*-5 (see Supplementary Fig. S3C at *JXB* online). Cd tolerance of AtABCC3ox-*abcc1abcc2*-1 and AtABCC3ox-*abcc1abcc2*-3 seedlings was assessed at 0 and 60 μM CdSO_4_ with or without β-oestradiol, in the presence or absence of 0.5mM BSO. After 9 d, seedling fresh weight and root length were analysed. As shown in Fig. 7D–F, a significant increase in both fresh weight and root length was observed in AtABCC3ox-*abcc1abcc2*-1 and AtABCC3ox-*abcc1abcc2*-3 seedlings grown with β-oestradiol compared with uninduced seedlings.

The increase in root length of AtABCC3ox-*abcc1abcc2*-1 was not observed in the presence of 0.5mM BSO (Fig. 7D–F), indicating that the observed BSO effect is specific for the transporter AtABCC3.

**Fig. 8.**
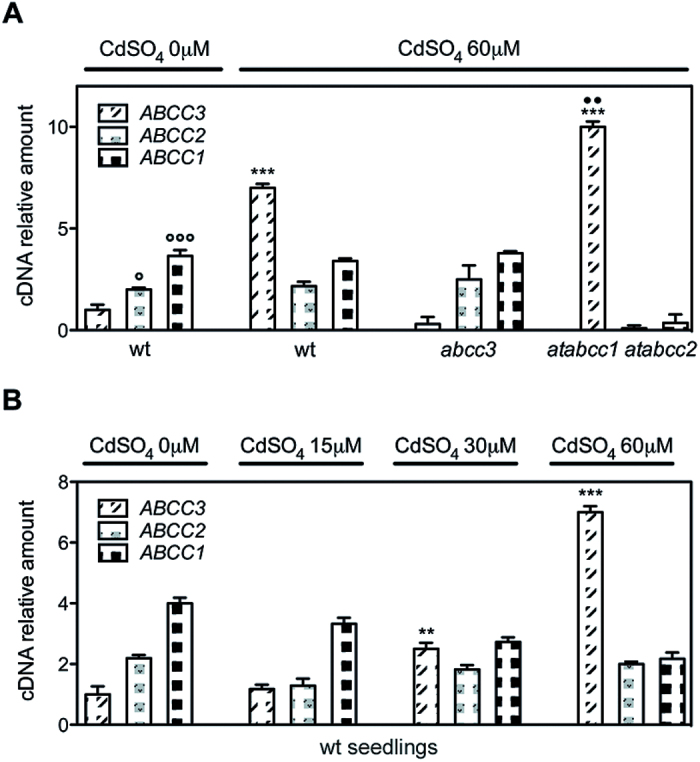
Comparative analysis of *AtABCC3*, *AtABCC2*, and *AtABCC1* transcript levels in wild type, *abcc3,* and *atabcc1 atabcc2* seedlings exposed to 60 μM CdSO_4_ and in wild type seedlings at different Cd concentrations. (A) Real-time RT–PCR of mRNA extracted from wild type, *abcc3*, and *abcc1abcc2* seedlings grown for 9 d at 0 or 60 μM CdSO_4_ (as indicated). Data are expressed as a mean value (*n*=3) of *AtABCC3*, *AtABCC1*, and *AtABCC2* cDNA levels relative to actin cDNA. Error bars indicate the SE. (B) Real-time RT–PCR of mRNA extracted from wild type seedlings grown for 9 d at 0, 15, 30, and 60 μM CdSO_4_. Data are expressed as a mean value (*n*=3) of *AtABCC3 AtABCC1*, and *AtABCC2* cDNA levels relative to actin cDNA. Error bars indicate the SE. Asterisks indicate a significant difference in *AtABCC3* transcript level from wild type seedlings at 0 μM CdSO_4_ (**P<0.01, ***P<0.001). Dots indicate a significant difference from *AtABCC3* transcript level in wild type seedlings at 60 μM CdSO_4_ (••*P* <0.01). Circles indicate a significant difference in *AtABCC1* and *AtABCC2* transcripts from the *AtABCC3* transcript level in wild type seedlings grown in the absence of Cd (°*P*< 0.05, °°°*P*< 0.001). wt, wild type.

To determine whether the relative transcript levels of *AtABCC1*, *AtABCC2*, and *AtABCC3* are consistent with the above-reported Cd tolerance of *abcc3* and *atabcc1 atabcc2* seedlings, a qRT-PCR analysis of mRNA extracted from wild type, *abcc3*, and *atabcc1 atabcc2* seedlings grown for 9 d at 0 or 60 μM CdSO_4_ was performed. As shown in Fig. 8A, in the absence of Cd the transcript levels of *AtABCC1* and *AtABCC2* were, respectively, 4- and 2-fold higher than that of *AtABCC3*. In contrast, at 60 μM CdSO_4_, the transcript levels of *AtABCC1* and *AtABCC2* did not increase, whereas the transcript level relative to *AtABCC3* increased by 6.9-fold, being 1.7- and 3.4-fold higher, respectively, than that of *AtABCC1* and *AtABCC2*. Interestingly, in *abcc3* mutants at 60 μM CdSO_4_, the transcript levels of *AtABCC1* and *AtABCC2* were comparable with those of wild type seedlings, whereas in *atabcc1 atabcc2* seedlings the level of *AtABCC3* transcript further increased, compared with that of wild type seedlings, being 3.2- and 6.8-fold higher, respectively, than that of *AtABCC1* and *AtABCC2*. The Cd-induced high level of *AtABCC3* transcript accounts for the slight differences in Cd sensitivity between *abcc3* and *atabcc1 atabcc2* seedlings at 60 μM CdSO_4_ (Fig. 7A–C).

To determine whether the relative slight differences in growth in the presence of Cd between *abcc3* and *atabcc1 atabcc2* mutants would be seen when Cd was added during the germination phase (see Park *et al.*, 2012), the same Cd tolerance assay was performed by incubating wild type, *abcc3*, and *atabcc1 atabcc2* seeds on a medium containing 60 μM CdSO_4_. As shown in Supplementary Fig. S4A at *JXB* online, after 14 d in the absence of Cd the growth of *abcc3* seedlings and that of wild type and *atabcc1 atabcc2* seedlings was comparable. In contrast, in the presence of 60 μM CdSO_4_, the growth of *abcc3* seedlings was similar to that observed when seeds were germinated without Cd, whereas that of *atabcc1 atabcc2* double mutants was severely inhibited in terms of root length (Supplementary Fig. S4A, B at *JXB* online). These data suggest that, in contrast to *AtABCC1* and *AtABCC2*, *AtABCC3* does not play a role in Cd tolerance during seed germination.

The relative transcript levels of *AtABCC1*, *AtABCC2*, and *AtABCC3* under these experimental conditions were evaluated by means of a qRT-PCR analysis of mRNA extracted from wild type seedlings 5 d after germination at 60 μM CdSO_4_. As shown in Supplementary Fig. S4C at *JXB* online, in the absence of Cd the levels of *AtABCC1* and *AtABCC2* transcripts were 4- and 2-fold higher, respectively, than that of *AtABCC3*, similar to what was described in the previous experiment (Fig. 8A), whereas in the presence of Cd the transcript levels of *AtABCC3* did not increase. The lack of Cd-induced *AtABCC3* expression during germination accounts for the dramatic differences in Cd sensitivity of *abcc3* and *atabcc1 atabcc2* seedlings under these experimental conditions.

As it is known that *abcc3* seedlings are not sensitive to low Cd concentrations (15 μM) and only slight sensitive to 30 μM CdSO_4_ (Fig. 1), to determine whether *AtABCC3* expression was induced at low Cd concentrations, the transcript level of *AtABCC3* was analysed at different Cd concentrations in comparison with that of *AtABCC1* and *AtABCC2.* A qRT-PCR analysis of mRNA extracted from wild type seedlings grown for 9 d at 0, 15, 30, or 60 μM CdSO_4_ was performed. As shown in Fig. 8B, the level of *AtABCC1* and *AtABCC2* transcripts in seedlings grown in the presence of all Cd concentrations was comparable with that in the absence of Cd. In contrast, while at 15 μM CdSO_4_ the *AtABCC3* transcript level was comparable with that in the absence of Cd, at 30 μM CdSO_4_ a slight but significant increase (~1.5-fold) was observed.

These data indicate that little expression of *AtABCC3* occurs at low Cd concentrations.

## Discussion

The ABC transporter AtABCC3 has for a long time been considered a good candidate for Cd transport into the vacuole as it partially complements the loss of the ABC protein YCF1 involved in Cd detoxification in *S. cerevisiae* (Tommasini *et al.*, 1998). Furthermore, *AtABCC3* expression is induced by Cd (Bovet *et al.*, 2003), and the AtABCC3 protein is localized in the vacuolar membrane (Dunkley *et al.*, 2006). However, the role of AtABCC3 in Cd tolerance and the substrates transported by AtABCC3 remained to be examined (Kang *et al.*, 2011).

Here, utilizing an *Arabidopsis* mutant deficient in AtABCC3 (*abcc3*), and plants overexpressing an inducible form of AtABCC3 in a wild type and in a PC-deficient mutant background, strong evidence is provided that AtABCC3 confers Cd tolerance by sequestering PC–Cd complexes in vacuoles.

In the overexpressor lines, the *AtABCC3* gene is under the control on a β-oestradiol-inducible promoter, allowing *AtABCC3* overexpression to be induced only when Cd was present in the medium. Seedling growth was evaluated by using two different parameters, fresh weight and root growth, as in Brunetti *et al.* (2011).

It is shown here that growth of *abcc3* mutant seedlings is hampered at any tested Cd concentration, except at very low concentrations which are not inhibitory for wild type seedlings. In agreement with this, *AtABCC3*-overexpressing plants show a slight but significantly higher root growth rate compared with the wild type at relatively high Cd concentrations. In contrast, no effects were observed at a lower Cd concentration, that causes just a slight reduction in wild type seedling growth. A possible explanation is that the Cd transport activity exerted by AtABCC3 is low at low Cd concentrations, as suggested by qRT-PCR analysis that shows a low level of *ABCC3* transcript at 30 μM CdSO_4_, and as previously shown for As transport by the ABCC transporters AtABCC1 and AtABCC2 when expressed in yeast (Song *et al.*, 2010). The present results on Cd tolerance of *abcc3* mutant seedlings are not in contrast to those presented by Park *et al.* (2012) where *abcc3* mutant seedling growth was shown to be comparable with that of wild type seedlings in the presence of Cd. The experimental conditions utilized by Park *et al.* (2012) were different from those used here, as seedlings were here exposed to Cd after germination. When seeds were germinated in the presence of Cd, results similar to those of Park *et al.* (2012) were obtained, as *abcc3* seedlings under those conditions show only a very slight Cd sensitivity.

By analysing the cytosolic and vacuolar Cd distribution in the *abcc3* mutant and in AtABCC3-overexpressing protoplasts, it is shown here that the effects of AtABCC3 on *Arabidopsis* Cd tolerance are due to its capacity to transport Cd into the vacuole. To distinguish between vacuolar and cytosolic Cd in protoplasts of the same lines, an innovative single-cell analysis was performed based on two different fluorochromes, BTC-5N and Leadmium™ Green AM dye. BTC-5N has been previously used to detect Cd in the cytosol of wheat root and shoot protoplasts (Lindberg *et al.*, 2004, 2007), while Leadmium™ Green AM dye has been used to detect Cd in the vacuole of *Arabidopsis* plant protoplasts (Park *et al.*, 2012) or to determine Cd distribution in entire organs, such as roots of two different *Sedum alfredii* ecotypes (Lu *et al.*, 2008). It is shown here that in protoplasts isolated from *abcc3* mutant lines there is a decrease in vacuolar Cd and a concomitant increase in cytosolic Cd compared with those of the wild type, whereas in AtABCC3ox protoplasts there is an increase in vacuolar Cd and a decrease in cytosolic Cd.

It is also shown that the total amount of Cd is not altered in all AtABCC3ox seedlings grown in the presence of Cd, under different experimental conditions. Similarly roots and shoots from the overexpressing lines have a Cd content similar to the wild type, suggesting that the transport of cytosolic Cd into the vacuole has no effect on total Cd accumulation in the cell.

Three lines of evidence based on the effects of *AtABCC3* overexpression indicate that this ABCC protein acts by transporting PC–Cd complexes into the vacuole. First, by overexpressing *AtABCC3* in *cad1-3* mutant lines defective in PC production (Howden *et al.*, 1995) no enhanced Cd tolerance was induced even when lines were exposed to low Cd concentrations. Secondly, by overexpressing *AtABCC3* in the presence of BSO, which prevents the accumulation of PCs by reversibly inhibiting the key enzyme in GSH biosynthesis, no enhanced Cd tolerance was induced. Lastly, *AtABCC3* overexpression in the *atabcc1 atabcc2* double mutant background defective in the PC–Cd transporters *AtABCC1* and *AtABCC2* (Park *et al.*, 2012) restores the Cd sensitivity of *atabcc1 atabcc2* double mutant seedlings, but not in the presence of BSO, indicating that BSO effects are specifically on *AtABCC3*.

By analysing the relative abundance of *AtABCC1*, *AtABCC2*, and *AtABCC3* transcripts at different Cd concentrations, it was shown that *AtABCC3* expression is regulated by Cd and that its activity is co-ordinated with the activity of AtABCC1 and AtABCC2. The constitutive level of *AtABCC3* is lower than that of *AtABCC1* and *AtABCC2* at low Cd concentrations (15 μM) and during seed germination, but its transcript level increases at high Cd concentration (60 μM), being higher than that of *AtABCC1* and *AtABCC2*. In addition a further increase of *AtABCC3* mRNA is observed in *atabcc1 atabcc2* double mutant seedlings exposed to high Cd concentrations, suggesting a compensative regulation of this Cd-inducible gene in the absence of *AtABCC1* and *AtABCC2*.

The results obtained are in accord with those of Park *et al.* (2012), who showed that the Cd-sensitive phenotype of the *atabcc1 atabcc2* double mutant defective in *AtABCC1* and *AtABCC2* PC–Cd transporters is not as severe as that of *cad1-3* (lacking PCs), suggesting that other transporter(s) may be able to compartmentalize PC–Cd complexes. Taken all together, these results indicate that in *Arabidopsis* several different ABCC PC–Cd transporters act in compartmentalizing Cd into the vacuole. This redundancy may be due to a lack of transporter specificity since all three proteins are involved in the transport of other xenobiotics/metabolites: AtABCC1 is involved in the transport of glutathione *S*-conjugates of xenobiotics and folate, while AtABCC2 and AtABCC3 are able to transport glutathione *S*-conjugates of xenobiotics and chlorophyll catabolites (Lu *et al.*, 1997; Frelet-Barrand *et al.*, 2008). Interestingly, while *AtABCC3* expression is induced by Cd (Bovet *et al.*, 2003; this study), thus ensuring a response related to Cd concentration or to PC–Cd complexes in the cell, *AtABCC1* and *AtABCC2* are constitutively expressed at a higher level and do not respond to Cd exposure. Furthermore, *AtABCC3* is part of a cluster—possibly due to gene duplication (Kolukisaoglu *et al.*, 2002)—of three Cd-regulated *ABCC/MRP* genes (*AtABCC6*, *AtABCC3*, and *AtABCC7*) localized in chromosome 3. A slight sensitivity to Cd has been described for *atabcc6* mutant seedlings (Gaillard *et al.*, 2008), while Park *et al.* (2012) report that root length was not altered in *atabcc6* seedlings at different Cd concentrations. On the other hand, an increase in Cd tolerance was observed by overexpressing *AtABCC7* in tobacco lines, while no Cd sensitivity was exhibited by *atabcc7* seedlings after exposure to Cd (Park *et al.*, 2012). Further work is therefore necessary to assess whether *AtABCC6* and *AtABCC7* are also involved in Cd tolerance as members of a Cd-inducible transport system.

In conclusion, the data indicate a substantial role for AtABCC3 in Cd detoxification whereby AtABCC3 detoxifies Cd by transporting PC–Cd complexes into the vacuoles, and that it can functionally complement *abcc1 abcc2* mutants. Further studies are needed to define whether AtABCC3 is also involved in tolerance to As and to other metals.

## Supplementary data

Supplementary data are available at *JXB* online.

Figure S1. Effects of β-oestradiol on wild type and AtABCC3ox seedling growth.

Figure S2. Cytosolic and vacuolar regions in wild type, *abcc3*, and AtABCC3ox protoplasts.

Figure S3. Quantitative analysis of *AtBCC3* in wild type, *cad1-3* and *abcc1 abcc2* lines overexpressing *AtABCC3*.

Figure S4. Cd tolerance of *abcc3* and *atabcc1 atabcc2* mutant seedlings exposed to Cd during the germination phase and quantitative analysis of *AtABCC3, AtABCC2*, and *AtABCC1* transcripts in wild type seedlings exposed to Cd during the germination phase

Supplementary Data
