# Supplementary material for: Cadmium-inducible expression of the ABC-type transporter AtABCC3 increases phytochelatin-mediated cadmium tolerance in Arabidopsis
Source: J Exp Bot. 2015 Apr 21;66(13):3815–29. doi: 10.1093/jxb/erv185 (PMC4473984; doi:10.1093/jxb/erv185)
Supplement: Supplementary Data [file supp_66_13_3815__index.html]

Cadmium-inducible expression of the ABC-type transporter AtABCC3 increases phytochelatin-mediated cadmium tolerance in Arabidopsis — Cadmium-inducible expression of the ABC-type transporter AtABCC3 increases phytochelatin-mediated cadmium tolerance in Arabidopsis — Supplementary Data 

# Cadmium-inducible expression of the ABC-type transporter *AtABCC3* increases phytochelatin-mediated cadmium tolerance in *Arabidopsis*

## Supplementary Data

Data files

**Files in this Data Supplement:**

- Supplementary Data - Supplementary Data
