# Supplementary material for: Cadmium-inducible expression of the ABC-type transporter AtABCC3 increases phytochelatin-mediated cadmium tolerance in Arabidopsis
Source: J Exp Bot. 2015 Apr 21;66(13):3815–29. doi: 10.1093/jxb/erv185 (PMC4473984; doi:10.1093/jxb/erv185)
Supplement: Supplementary Data [file supp_erv185_jexbot143966_file001.pdf]

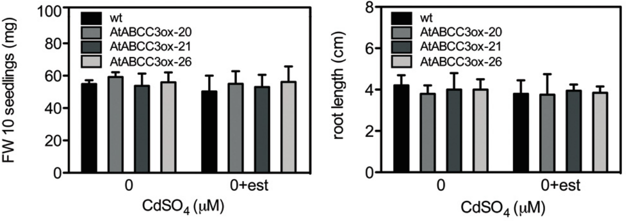

Figure S1

Effects of β-estradiol on wild type and AtABCC3ox seedling growth.

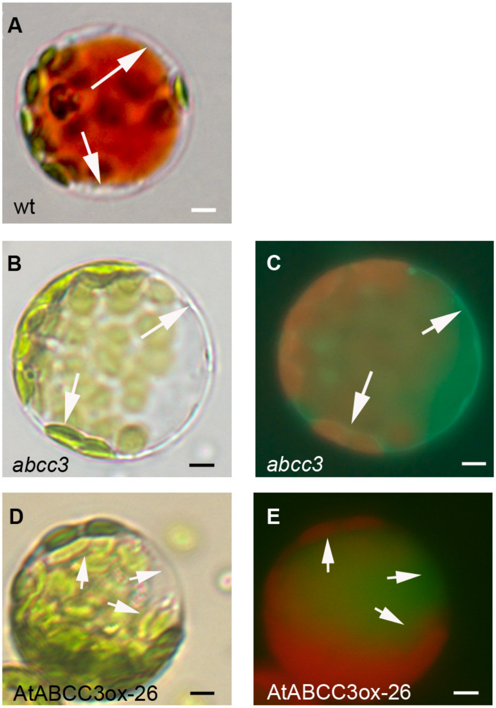

Figure S2

Cytosolic and vacuolar regions in wild type, *abcc3* and AtABCC3ox protoplasts.

A) A protoplast from wild type leaves stained with neutral red. The vacuole is in red colour. (B,C) Leaf protoplasts from *abcc3* leaves, loaded with BTC-5N. White-light image (B), and fluorescent image with the bright green signal in the cytosol (C).

(D,E) Protoplasts from AtABCC3ox-26 leaves loaded with leadmium green. White-light image (D), and fluorescent image with the fluorescent green signal in the vacuole (E). The arrows show the tonoplast. Bars = 10 μm.

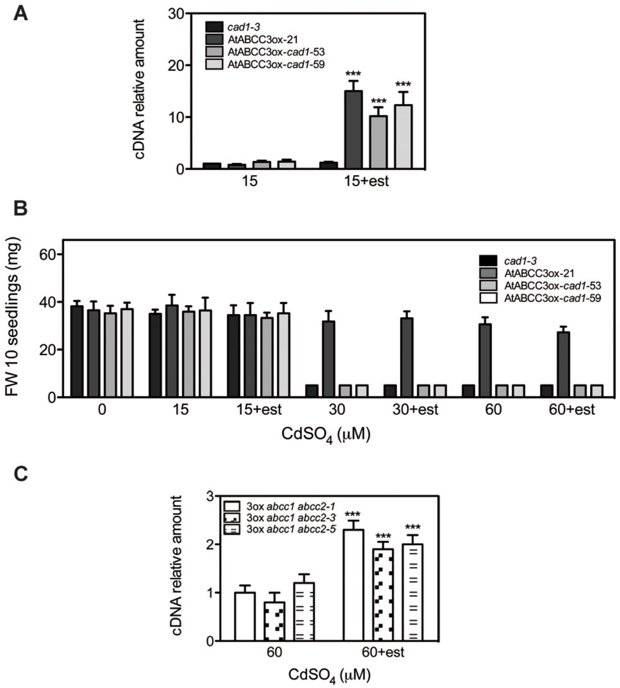

Figure S3

Quantitative analysis of *AtBCC3* in *cad1-3,* and in wild type, *cad1-3* and *abcc1abcc2* lines overexpressing *AtABCC3*.

A) *AtBCC3* transcript levels in *cad1-3,* AtABCC3ox-21, AtABCC3ox-*cad1*-53 and AtABCC3ox-*cad1*-59 seedlings grown for 9 days in the absence or presence of β-estradiol. Error bars indicate SE. B) Fresh weight of AtABCC3ox-*cad1*-53 and AtABCC3ox-*cad1*-59 seedlings grown for 9 days at 15, 30, and 60 μM $CdSO_4$, in the absence or presence of β-estradiol. Values correspond to means (*n*=3). C) *AtABCC3* transcript levels in AtABCC3ox-*abcc1abcc2-1,* AtABCC3ox-*abcc1abcc2-3* and AtABCC3ox-*abcc1abcc2-5* seedlings grown for 9 days in the absence or presence of β-estradiol. Error bars indicate SE. Asterisks indicate a significant difference from seedlings grown in the absence of β-estradiol (***P<0.001).

3ox, AtABCC3ox; est, β-estradiol.

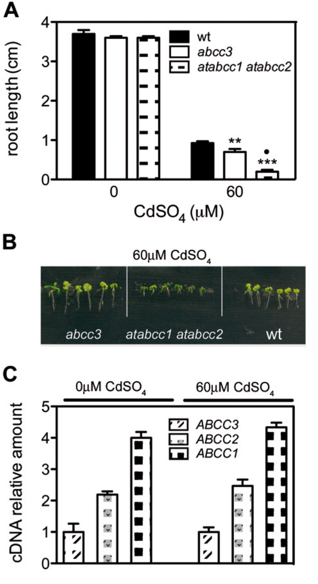

Figure S4

Cd tolerance of *abcc3* and *atabcc1 atabcc2* mutant seedlings exposed to Cd during the germination phase (A,B) and quantitative analysis of *AtABCC3, AtABCC2* and *AtABCC1* transcripts in wild type seedlings exposed to Cd during the germination phase (C). Values correspond to means (*n*=3). Error bars indicate SE. Asterisks indicate a significant difference from wild type grown in the presence of 60 mM $CdSO_4$ (**P<0.01, ***P<0.001). A single dot indicates a significant difference from *abcc3* roots grown at 60 mM $CdSO_4$ (•P <0.05). wt, wild type.
